# Comprehensive Approaches to Endometriosis Management

**DOI:** 10.3390/jcm14124324

**Published:** 2025-06-17

**Authors:** Arrigo Fruscalzo, Michael D. Mueller, Gaby Moawad, Attila Bokor, Horace Roman, Carolin Marti, Benedetta Guani, Jean-Marc Ayoubi, Anis Feki

**Affiliations:** 1 Department of Gynecology and Obstetrics, HFR, University Hospital of Fribourg, 1708 Fribourg, Switzerland; carolin.marti@h-fr.ch (C.M.); benedetta.guani@h-fr.ch (B.G.); anis.feki@h-fr.ch (A.F.); 2 Department of Gynecology and Obstetrics, University Hospital of Berne, 3010 Bern, Switzerland; michel.mueller@insel.ch; 3 Department of Obstetrics and Gynecology, The George Washington School of Medicine and Health Sciences, Washington, DC 20052, USA; drgabymoawad@iceaps.com; 4 The Center for Endometriosis and Advanced Pelvic Surgery, Miami, FL 33133, USA; 5 Department of Obstetrics and Gynecology, Semmelweis University, 1088 Budapest, Hungary; attila.z.bokor@gmail.com; 6 Institut Franco-Européen Multidisciplinaire d’Endométriose, 33000 Bordeaux, France; horace.roman@gmail.com; 7 Department of Gynecology and Obstetrics and Reproductive Medicine, Hôpital Foch, 75017 Paris, France; jm.ayoubi@hopital-foch.org

## 1. Editorial

Endometriosis is a debilitating disease that affects millions worldwide. Despite its benign nature, it brings heavy clinical, social, health, and financial burdens, primarily due to its elusiveness and chronic development pathway [1]. Although much research is being carried out, diagnosis and treatment options remain a challenge. Thus, a bold new frontier is waiting to be explored regarding the way we approach and treat this disease.

This Special Issue collates eight manuscripts covering topics related to endometriosis, providing an overview of the current evidence-based knowledge on the disease. A detailed list of contributions is provided at the end of this article.

Two of these studies cover the issue of psychological and mental health in chronic diseases such as endometriosis. This disease is a chronic pain-associated disorder that is often linked to social impairment and mental disorders, necessitating psychiatric support [2]. There is evidence that this burden can affect women to the extent that it induces specific neurological patterns, the so-called “endometriosis brain” [3]. Addressing this topic is of paramount importance in order to fill the knowledge gap and improve the management of this too often neglected issue. In Contribution 1, the authors carry out a comprehensive narrative review on the psychosomatic burden in endometriosis. Similarly, in Contribution 2, the authors tackle the problem of mental health and quality of life in adolescents with this disease, showing that adjuvant hormonal therapy with dienogest after surgery significantly reduces pain symptoms.

Regarding endometriosis-related comorbidities, one greatly pertinent research area is the potential impact of a specific immunologic fingerprint of endometriosis [4]. There is decent epidemiological evidence for a link between endometriosis and several autoimmune diseases, including systemic lupus erythematosus, Sjögren’s syndrome, rheumatoid arthritis, autoimmune thyroid disorder, coeliac disease, inflammatory bowel disease, Addison’s disease, and multiple sclerosis [5]. However, the mechanisms underpinning the occurrence of these disorders are still poorly understood [6]. In this context, one innovative paper in this Special Issue investigates a potential common immune pattern linking patients affected by multiple sclerosis and endometriosis (Contribution 3). The authors found specific immune features that were characteristic of patients affected by endometriosis and multiple sclerosis, most notably increased levels of regulatory T cells (Tregs). Similarly, in Contribution 4, the authors investigated the relationship between temporomandibular joint symptoms and pelvic pain in endometriosis; they revealed a correlation between the two, highlighting the need for further research in the area of endometriosis and musculoskeletal pain.

The other contributions mainly deal with infertility, another major matter of concern in endometriosis. While it is estimated that endometriosis affects approximately 10% of reproductive-age women, its prevalence in infertile women is expected to be much higher, ranging from 30% to 50% [7]. Despite this evidence, a clear physio-pathological explanation of this pattern remains elusive. Many factors seem to play a role, including an altered pelvic anatomy, disrupted ovarian reserve/function, and compromised endometrial receptivity, as well as systemic negative effects related to chronic inflammation [8]. Contribution 5 presents data confirming the negative impact of adenomyosis on the clinical outcomes of patients undergoing frozen embryo transfer (FET): links to higher miscarriage rates and lower clinical pregnancy and live birth rates. Another study explores the impact of endometriosis on the composition of follicular fluid in IL6 and AMH levels (Contribution 6). In addition to these original data, Contributions 7 and 8 are two comprehensive overviews on fertility preservation related to the ovarian reserve in case of surgery for endometriosis.

## 2. Future Outlooks

The contributions presented in this Special Issue offer a snapshot of current hot topics in endometriosis research. Looking ahead, future studies should focus on developing strategies to improve patients’ quality of life, reduce diagnostic delays, and address the stigma associated with this condition. Particular attention should be paid to the broader impact of endometriosis, including its social burden, as well as its effects on women’s mental health and physical well-being.

New strategies for both the medical and surgical treatment of endometriosis are urgently needed; a deeper understanding of the pathogenesis of the disease will be key to achieving this goal. Such knowledge will support the development of more targeted medical therapies and enable the refinement of surgical techniques tailored to individual patient needs. Moreover, a more integrative, multidisciplinary approach should be a cornerstone in advancing treatment options.

Reproductive health remains a critical area in endometriosis research, as both the condition itself and its treatments can significantly impact fertility. Greater efforts are needed to develop improved strategies for fertility preservation and the management of endometriosis-related infertility. For instance, new insights into how endometriosis affects ovarian function and embryo implantation could enhance current approaches to reproductive planning and assisted reproductive technologies.
